# Clinical Notes

**Published:** 1892-03

**Authors:** 


### CLINICAL NOTES.

(Translated from *Revistá Homœopaticá*, April, 1891.)

Importance of Moral Symptoms.—It is true that homœopathic doctors explicitly or tacitly give importance to moral symptoms, nevertheless we do not believe that we give them their full value. Hahnemann, whose advice it is necessary to so often refer to, says: "The moral study of the patient is the most sure and decisive guide for the selection of the homœopathic remedy." To better illustrate this, Aconite very rarely or never effects a lasting cure when the patient is tranquil, or Nux-vomica when the disposition is gentle and phlegmatic. Pulsatilla—lively, serene, or fierce. Ignatia for strong minds and but little susceptible to fear or pain. With the necessary attention to these seeming smallnesses but very useful pathologically the homœopathic doctor will effect the greatest cures.

Calcarea Phosphorica in Gonorrhœa.—This insidious disease was cured by Dr. Berridge in a man who had it the second time. He showed no characteristic symptoms, and after having tried almost fruitlessly Merc., Canth., Nitric acid, Thuja, Kali-bicrom., Argent-nit., Cann., Nux—Calc-phos. was used with the very best results, guided by the following characteristics: He had painful erections when traveling on railroad cars, excepting when he found himself obliged to enter into conversation, and as this symptom corresponds to the said remedy he gave him seven doses to take once a day of Fincke CM. The result was rapid and the disagreeable symptom and all remains of gonorrhœa disappeared. I. N. R.

